# Development of Machine Learning Systems to Predict Cancer-Related Symptoms With Validation Across a Health Care System

**DOI:** 10.1200/CCI-25-00073

**Published:** 2025-09-25

**Authors:** Baijiang Yuan, Muammar Kabir, Jiang Chen He, Yuchen Li, Benjamin Grant, Sharon Narine, Mattea Welch, Sho Podolsky, Ning Liu, Rami Ajaj, Luna Jia Zhan, Aly Fawzy, Janine Xu, Yuhua Zhang, Vivien Yu, Wei Xu, Rahul G. Krishnan, Steven Gallinger, Kelvin K.W. Chan, Monika K. Krzyzanowska, Tran Truong, Geoffrey Liu, Robert C. Grant

**Affiliations:** ^1^ Division of Medical Oncology and Hematology, Princess Margaret Cancer Centre, University Health Network, Toronto, Canada; ^2^ Institute of Medical Science, University of Toronto, Toronto, Canada; ^3^ Cancer Digital Intelligence, University Health Network, Toronto, Canada; ^4^ Ontario Institute for Cancer Research, Toronto, Canada; ^5^ Institute for Clinical Evaluative Sciences, Toronto, Canada; ^6^ Biostatistics Department, University Health Network, Toronto, Canada; ^7^ Department of Computer Science, University of Toronto, Toronto, Canada; ^8^ Department of Laboratory Medicine and Pathobiology, University of Toronto, Toronto, Canada; ^9^ Vector Institute, Toronto, Canada; ^10^ Odette Cancer Center, Sunnybrook Health Sciences Center, Toronto, Canada

## Abstract

**PURPOSE:**

Cancer and its treatment cause symptoms. In this study, we aimed to develop machine learning (ML) systems that predict future symptom deterioration among people receiving treatment for cancer and then validate the systems in a simulated deployment across an entire health care system.

**METHODS:**

We trained and tested ML systems that predict a deterioration in nine patient-reported symptoms within 30 days after treatments for aerodigestive cancers, using internal electronic health record (EHR) data at Princess Margaret Cancer Centre (3,229 patients; 20,267 treatments). The primary performance metric was the area under the receiver operating characteristic curve (AUROC). The best-performing systems in the held-out internal test set were then externally validated across 82 cancer centers in Ontario (12,079 patients; 77,003 treatments) by adapting techniques from meta-analysis.

**RESULTS:**

The best ML systems predicted symptom deterioration with AUROCs ranging from 0.66 (95% CI, 0.63 to 0.69) for dyspnea to 0.73 (95% CI, 0.71 to 0.75) for drowsiness in the internal test cohort. Treatments flagged as high-risk were significantly associated with future symptom deterioration (odds ratios [ORs], 2.53-6.56; all *P* < .001) and emergency department visits for dyspnea (OR, 1.85; *P* = .008), depression (OR, 1.84; *P* = .04), and anxiety (OR, 2.66; *P* < .001). In the external validation cohort, the AUROCs for different symptoms meta-analyzed across centers ranged from 0.67 (95% CI, 0.66 to 0.68) to 0.73 (95% CI, 0.72 to 0.74). Performance across centers displayed significant heterogeneity for six of nine symptoms (I^2^, 46.4%-66.9%; *P* = .004 for dyspnea, *P* < .001 for the rest).

**CONCLUSION:**

ML can predict future symptoms among people with cancer from routine EHR data, which could guide personalized interventions. Heterogeneous performance across centers must be considered when systems are deployed across a health care system.

## INTRODUCTION

Improving quality of life is a fundamental goal of cancer care.^1,2^ The presence, severity, and functional impact of symptoms contribute to the quality of life experienced by people with cancer.^3,4^ Unfortunately, most people with cancer experience moderate to severe symptoms after diagnosis.^5^ Furthermore, symptoms are the most common reason for acute care in the emergency room or hospital,^6^ which constitutes approximately half of cancer-related costs.^7^ Innovative strategies to reduce the burden of symptoms among people with cancer are urgently needed.

**CONTEXT**

**Key Objective**
Can machine learning (ML) predict future symptom deteriorations among patients receiving systemic therapy for cancer?
**Knowledge Generated**
Our study demonstrates that ML can identify patients who are at high risk of symptom deterioration after systemic therapy for cancer, which could be used to guide preventive interventions. The ML systems displayed heterogeneous performance across 82 cancer centers during external validation, suggesting that infrastructure for local performance monitoring and fine-tuning is required to deploy systems across health care systems.
**Relevance *(J.L. Warner)***
This study demonstrates the feasibility of identifying patients at high risk of worsening symptoms in the near-term, using ML. Modest performance as measured by AUC was mostly preserved in external validation, and the observed performance could reflect limitations in the underlying source data, which were limited to routinely collected tabular electronic health record data.*
*Relevance section written by *JCO CCI* Editor-in-Chief Jeremy L. Warner, MD, MS, FAMIA, FASCO.

Symptom management is currently mostly reactive. For example, consensus guidelines focus on managing symptoms after they occur, depending on severity,^8-10^ except for nausea and vomiting,^11^ where prophylactic antiemetics are typically prescribed based on the risk of the chemotherapy regimen. Proactive strategies to prevent symptoms *before* they occur or worsen offer an opportunity to improve the quality of life for people with cancer.

Personalized prevention of symptoms requires identifying people at risk. Although early evidence supports that machine learning (ML) systems can predict future symptoms, existing studies are limited, considering only a single time point or lacking external validation.^12-14^ Whether future symptoms can be reliably predicted among patients with cancer longitudinally at the point of care remains an open question. Furthermore, clinical ML trained in one setting may not generalize to others.^15,16^

In this study, we developed and evaluated predictive ML systems that used routinely collected electronic health record (EHR) data to learn whether future cancer-related symptoms can be predicted to personalize preventive care. To understand how ML systems would scale across an entire health care system, we conducted an external validation across 82 cancer centers in Ontario.

## METHODS

**FIG 1.**
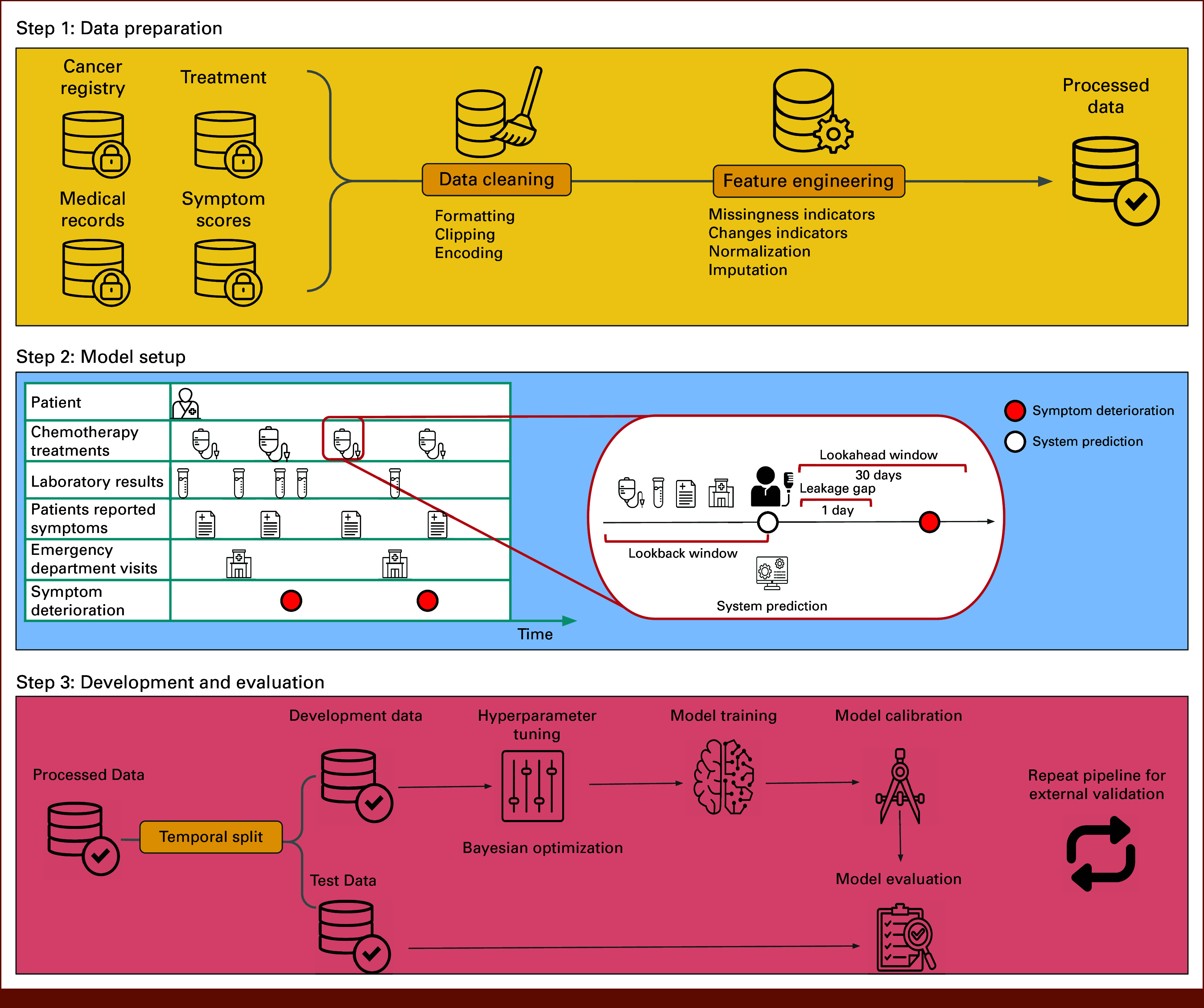
Study schema. The lookback windows for different features were 5 days for laboratory features, 30 days for patient-reported symptoms, and 5 years for emergency department visit data. Icons used in this figure are credited to their respective creators on The Noun Project^42^ under CC BY 3.0. These include works by SmashiconsGB (Lock Database), Adrian Fanani (Data cleaning), Hat-TechPK (Data engineering), Scarlett Mckay and Pixelz Studio (Patient), DARAYANI (Infusion), Cherry (Test tube), Vectorstall PK (Medical), yus (Hospital), Siti Solekah (System), DinosoftLabs (Volume control), Angela (Machine learning), Sutriman ID (Calibration), and Gregor Cresnar (Repeat). Adapted from He et al.^43^

Our study adhered to the TRIPOD Statement^17^ and was approved by the relevant Research Ethics Boards (nos. 06-0639, 19-5099, 037-2020). The overall study schema is depicted in Figure 1. The Data Supplement contains more detailed information.

### Cohorts

#### Internal Development and Test Cohorts

We developed and tested the ML systems using EHR data from the Princess Margaret Cancer Centre (PM), a large academic cancer center in Toronto, Canada. Patients were identified from the Princess Margaret Cancer Registry. We included patients with aerodigestive cancers because they are associated with a high burden of various symptoms and are the disease sites where the coauthors have clinical expertise. Patients were included if they received intravenous systemic therapy between January 1, 2014, and December 31, 2019. This internal cohort was divided into a development cohort with patients whose first treatments occurred from January 1, 2014, to September 30, 2017, and a test cohort for those who started after October 1, 2017. The development cohort was divided randomly 4:1 at the patient level into training and tuning cohorts.

### Predictive Features

We selected predictive features based on input from the medical oncologists on our team, literature review, and availability within the EHR. Features included demographics (age, sex, height, weight, and body surface area), cancer diagnosis (morphology and topography in International Classification of Diseases-O-3 codes),^18^ treatment (regimen type, intent, dosing, number of cycles, and days since starting and last treatment),^19^ laboratory test values (hematology and biochemistry), history of acute care use,^20^ and previous patient-reported symptoms^12,14^ (Data Supplement, Table S1).

### Targets

The target of the ML systems was to predict future deterioration in self-reported symptoms using the Edmonton Symptom Assessment Scale (ESAS).^21^ ESAS requires patients to rate nine symptoms on 10-point Likert scales. These symptoms include nausea, appetite, pain, dyspnea, fatigue, drowsiness, depression, anxiety, and well-being. ESAS scores are often categorized into none (0), mild (1-3), moderate (4-6), and severe (7-10).^22-24^ Therefore, to capture major symptom changes, we defined our primary target as an increase of three or more points in an ESAS symptom score within 30 days following each treatment session. Sensitivity analyses evaluated both a one-point increase, previously identified as the minimal clinically important difference,^25^ and an increase of at least four points. Symptom correlations were measured accounting for repeated measures.^26^

### Machine Learning Model Development

The system longitudinally evaluates patients before each treatment to predict symptom deterioration in the following 30 days. Treatment sessions followed by symptom deterioration the next day were excluded from the training to encourage the system to focus on detecting early warning signs rather than impending deterioration. Additionally, treatment sessions where specific symptoms were already severe (ESAS, 7-10) were excluded from training and testing, focusing only on patients at risk of future symptom deterioration. We trained various ML systems as described in the Data Supplement.

### System Evaluation

Our primary metric for evaluating performance was the area under the receiver operating characteristic curve (AUROC), which measured the ability of the systems to discriminate between high- and low-risk patients. The secondary metric of discrimination was the area under the precision-recall curve (AUPRC). The calibration of predictions was assessed using calibration curves and the mean and maximum calibration errors.^27,28^ Decision curve analysis evaluated the clinical utility of the system to guide interventions.^29^ To understand what features contribute to the predictions, we calculated Shapley additive explanation (SHAP) values^30^ for each feature for each target in the test cohort.

### Simulated Deployment as an Early Warning System

In practice, symptom prediction systems can be deployed with alarms to alert clinicians when patients face an elevated risk of symptom deterioration to prompt consideration of preventive interventions. We defined the alarm rate as the proportion of treatment sessions associated with an alarm. We initially considered a 10% alarm rate, which the medical oncologists on the team felt would balance alarm fatigue and time constraints while still identifying high-risk patients. We report the positive predictive value (PPV) and the sensitivity of alarms. Odds ratios (ORs) comparing the risk of symptom deterioration between treatments with and without alarms were estimated as a fixed effect in a generalized mixed-effect model, incorporating a linear fixed time effect and random patient effects to account for the longitudinal nature of the data. *P* values were used to test whether the ORs differed significantly from 1 at an alpha level of 0.05, with adjustments for multiplicity using the Bonferroni correction.

To account for the simultaneous prediction of multiple symptoms, we considered three alarm strategies: (1) the alarm rate for each symptom was 10%, irrespective of alarms for other symptoms (independent allocation); (2) the total alarm rate across all symptoms was 10%, allocating alarms equally across all symptoms so that each has an individual alarm rate of 1.11% (alarm-based allocation); and (3) the total alarm rate across all symptoms was 10%, applying the same risk threshold equally across all symptoms (risk-based allocation). We also considered the 5% and 15% alarm rates in sensitivity analysis.

### Association Between Alarms and Emergency Department Visits

Since symptoms are a major cause of acute care, we tested whether treatments with alarms for future symptoms were also associated with emergency department (ED) visits. The risk of ED visits within 30 days after treatment was compared for treatments with alarms versus treatments without alarms for each symptom using generalized linear mixed-effect models. *P* values were adjusted using the Bonferroni correction.

### External Validation

To assess how ML systems trained at a large urban academic cancer center would generalize to other cancer centers across the health care system, we tested the performance of the system at 82 other cancer centers in Ontario between October 1, 2017, and December 31, 2019. External validation used population-level data held at the Institute for Clinical Evaluative Sciences, which captures all health care services within the universal health care system in Ontario.^31^ We calculated the test AUROC of each symptom prediction system at each cancer center and reported the mean, IQR, and range. We estimated center-level variance in the AUROC using bootstrapping, tested for between-center heterogeneity in AUROC across centers using Cochran's Q with alpha of 0.1, and measured heterogeneity using I^2^ statistics.^32^ We also estimated the Pearson correlation coefficient between center-level attributes and the test AUROC at each center to determine whether any attributes were significantly associated with system validation. *P* values tested whether the correlation coefficients differed significantly from 0 at an alpha of 0.05 after adjustment for multiplicity.

## RESULTS

### Cohort

The internal EHR development cohort consisted of 2,269 patients who received 14,697 treatment sessions, with data spanning 218 features (Table 1, Data Supplement, Fig S1 and Table S1). Of the cohort, 948 (41.7%) were female, and the median age was 64 years with an IQR of 14 years. The most common primary cancer sites were lung (731, 32.3%), pancreatic (382, 16.8%), and colon cancers (189, 8.3%), reflecting the large pancreatic cancer program at the institution.^33^ The most common systemic regimens were fluorouracil, leucovorin, irinotecan, and oxaliplatin (217, 9.6%); infusional fluorouracil, leucovorin, and oxaliplatin (198, 8.7%); and fluorouracil, leucovorin, and irinotecan with bevacizumab (171, 7.5%) (Table 1). Among the nine symptoms, deterioration of three or more points on the ESAS scale occurred after 7.7%-13.7% of treatments and at some point during treatment for 24.8%-40.4% of patients. Symptoms were missing before or after treatment for 41.4%-42.2% of treatments. In cases where symptoms were reported before treatment, 24%-24.2% of treatments lacked symptom data after treatment (Data Supplement, Table S2). The internal test cohort displayed similar characteristics (Data Supplement, Table S3). Characteristics were generally similar between patients who did and did not report symptoms (Data Supplement, Table S4). Deterioration was correlated between different symptoms (correlation coefficients, 0.12-0.49), with drowsiness-fatigue and anxiety-depression showing the strongest associations (Data Supplement, Fig S2).

**TABLE 1.** Characteristics of the Treatment Session and Patients in the Internal Electronic Health Record–Based Development and Testing Cohorts

Characteristic	Development (January 1, 2014, to September 29, 2017)		Testing (October 2, 2017, to December 19, 2019)	
	Patients (N = 2,269)	Treatment Sessions (N = 14,697)	Patients (N = 960)	Treatment Sessions (N = 5,570)
Number of treatments, median (IQR)	4 (2-8)		4 (2-7)	
Age in years, median (IQR)	64 (56-70)	64 (56-70)	64 (57-71)	64 (57-71)
Female, No. (%)	948 (41.7)	6,304 (42.9)	372 (38.8)	2,183 (39.2)
Regimen, No. (%)^a^				
FOLFIRINOX	217 (9.6)	1,239 (8.4)	67 (7)	430 (7.7)
mFOLFOX6	198 (8.7)	954 (6.5)	61 (6.4)	256 (4.6)
FOLFIRI with bevacizumab	171 (7.5)	1,607 (10.9)	20 (2.1)	198 (3.6)
Cancer type, No. (%)^a^				
Lung	731 (32.2)	3,899 (26.5)	366 (38.1)	1,936 (34.8)
Pancreas	382 (16.8)	3,391 (23.1)	130 (13.5)	1,193 (21.4)
Colon	189 (8.3)	1,685 (11.5)	63 (6.6)	443 (8)
Symptoms deteriorations, No. (%)^a^				
Drowsiness	932 (41.1)	1,383 (9.4)	382 (39.8)	541 (9.7)
Fatigue	929 (40.9)	964 (6.6)	360 (37.5)	339 (6.1)
Appetite	904 (39.8)	1,203 (8.2)	349 (36.4)	459 (8.2)

Abbreviations: FOLFIRI, fluorouracil, leucovorin, and irinotecan; FOLFIRINOX, fluorouracil, leucovorin, irinotecan, and oxaliplatin; mFOLFOX6, modified fluorouracil, leucovorin, and oxaliplatin.

a Deterioration is defined as three or more points on the Edmonton Symptom Assessment Scale. We present the top three symptoms by patient frequency in the development cohort, with a full list in the Data Supplement (Table S2).

### System Development

**FIG 2.**
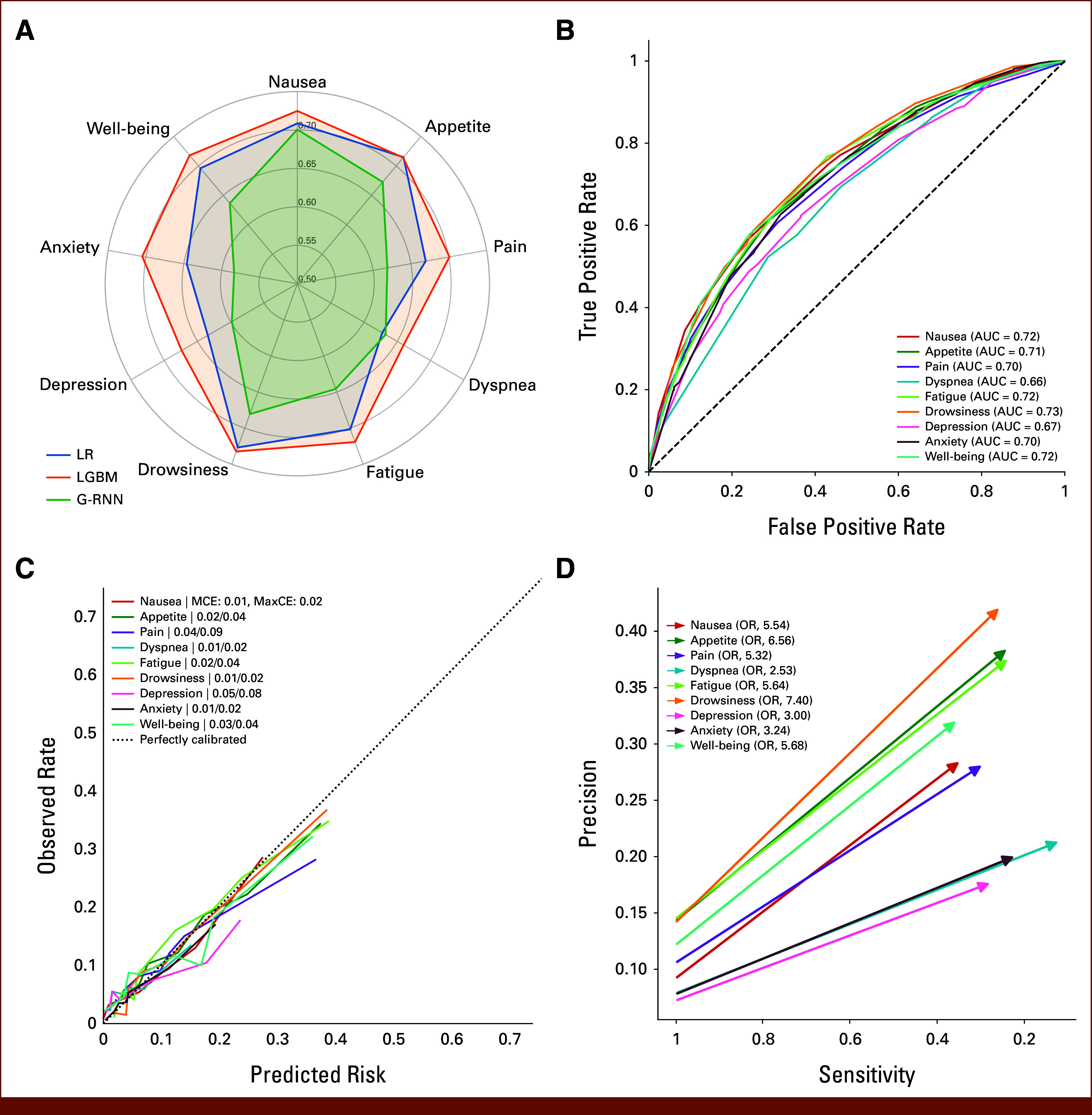
System performance in the internal test cohort. (A) Radar plots compared the areas under the receiver operating characteristic curve for LR, LGBM, and TCN. (B) Receiver operating characteristic curve for LGBM. (C) Calibration curve with isotonic regression for LGBM. (D) Arrow plot: Starting with 100% sensitivity and the event prevalence, corresponding to providing an alert before every treatment, the arrows point to the sensitivity and positive predictive value when the system is set to alert before 10% of treatments for each symptom. ORs compared symptom deterioration after treatments with alarms with treatments without alarms. LGBM, light gradient-boosting machine; LR, ridge logistic regression; MaxCE, maximum calibration error; MCE, minimum calibration error; OR, odds ratio; TCN, multi-task temporal convolutional neural network.

The LightGBM model showed the strongest performance in the internal tuning cohort for all symptoms, with a mean AUROC of 0.75 (95% CI, 0.74 to 0.76; Data Supplement, Tables S5-S13) across symptoms. The simpler logistic regression model demonstrated inferior performance, achieving a mean AUROC of 0.73 (95% CI, 0.72 to 0.74). We did not observe further improvement with longitudinal deep learning or multitask models (Fig 2A; Data Supplement, Table S14). Consequently, LightGBM was chosen as the ML system for downstream analysis.

Within the internal test cohort, the system predicted symptom deterioration with an AUROC ranging from 0.66 (95% CI, 0.63 to 0.69) for dyspnea to 0.73 (95% CI, 0.71 to 0.75) for drowsiness, achieving a mean AUROC of 0.71 (95% CI, 0.70 to 0.71) (Fig 2B; Data Supplement, Table S15), and AUPRC ranging from 0.15 (95% CI, 0.12 to 0.18) for dyspnea to 0.31 (95% CI, 0.28 to 0.35) for appetite (Data Supplement, Fig S3a and Table S16); 90% of predictions were under the 0.4 threshold (Data Supplement, Fig S3b). The system's mean calibration error ranged from 0.01 to 0.05, and the maximum calibration error ranged from 0.02 to 0.08 across all symptoms (Fig 2C).

In sensitivity analyses, the system demonstrated consistent performance, measured by AUROC, when predicting one-, three-, and four-point deteriorations (Data Supplement, Tables S17 and S18). AUPRC was higher for predicting one-point deteriorations, reflecting a higher baseline event rate.

Decision curve analysis showed that the system would provide a net benefit for guiding preventive intervention across various threshold probabilities when compared with intervening in all or none of the patients (Data Supplement, Fig S4). The narrowest range of threshold probabilities was 0.23-0.30 for depression, whereas the widest range was 0.07-0.42 for fatigue.

**FIG 3.**
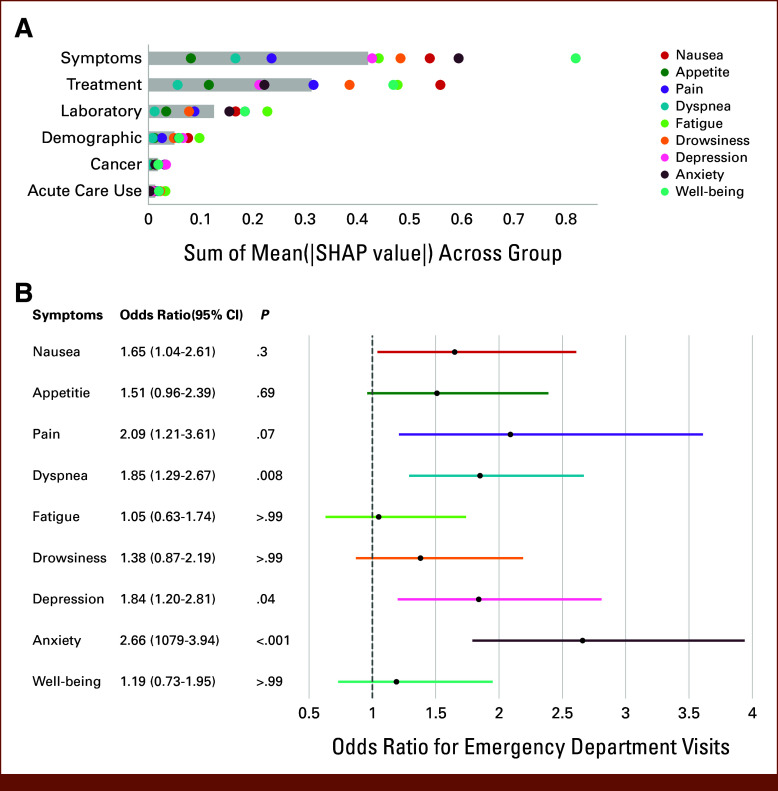
(A) The importance of groups of features predictions in the internal test cohort, measured using the sum of mean absolute SHAP values for each symptom (colored dots) and all symptoms (gray bars). (B) Odds ratios for the risk of emergency department visits within 30 days after an alarm. SHAP, Shapley additive explanation.

The systems generated predictions using different features for different symptoms based on SHAP values (Data Supplement, Figs S5 and S6). Symptoms, treatment, and laboratory were identified as the three most important groups of features (Fig 3A).

### Simulated Deployment as a Warning System

To understand how simultaneously predicting multiple outcomes may affect the clinical team, we simulated the deployment of the system using alarms. At the 10% alarm rate per symptom, alarms were significantly associated with future symptom deterioration (ORs, 2.53-7.40 comparing treatments with and without alarms; *P <* .001 for all symptoms; Fig 2D). For example, in the case of nausea, the 10% alarm rate occurred at the 22.9% risk threshold. Alarms had a PPV of 28.5% against a baseline prevalence of 9.2% (OR, 5.54 [95% CI, 4 to 7.68]; *P* < .001) and a sensitivity of 34.5%. Results were comparable with 5%, 10%, and 15% alarm rates per symptom (Data Supplement, Tables S19 and S20).

A 10% alarm rate *per symptom* led to alarms preceding 35.7% of treatments overall. We compared two strategies to restrict the overall alarm rate to 10% to reduce alarm fatigue across multiple predictions. Under alarm-based allocation, each symptom had a 1.11% alarm rate, with increased PPV and reduced sensitivity (Data Supplement, Fig S7a). Under risk-based allocation, a 41% risk threshold was applied equally across all symptoms, leading to a combined alarm rate of 10%. PPV increased for seven of nine symptoms (Data Supplement, Fig S7b). Notably, under risk-based allocation, the system would not generate any alarms for symptoms with the lowest rates of deterioration: anxiety and nausea (Data Supplement, Table S21).

### Association Between Alarms and Emergency Department Visits

We further evaluated whether the system could identify symptoms that lead to ED visits. At the 10% alarm rate under independent allocation, ED visits were significantly more common after alarms for three of the nine symptoms: dyspnea (OR, 1.85 [95% CI, 1.29 to 2.67]; *P* = .008), depression (OR, 1.84 [95% CI, 1.20 to 2.81]; *P* = .04), and anxiety (OR, 2.66 [95% CI, 1.79 to 3.94]; *P* < .001) (Fig 3B).

### External Validation Across the Health Care System

**FIG 4.**
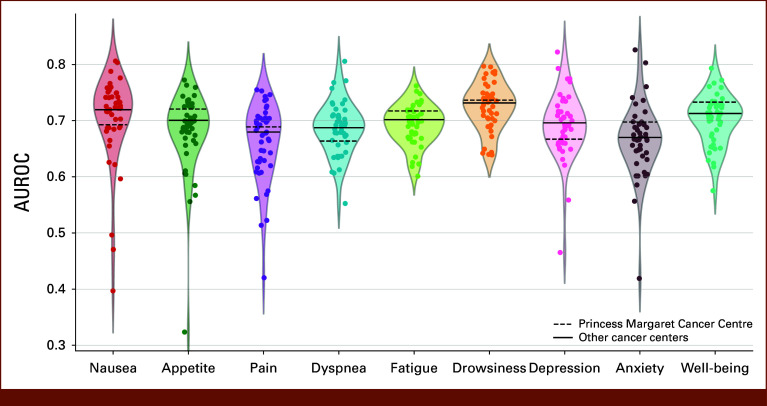
External validation of the system to predict symptom deterioration trained at Princess Margaret Cancer Centre, tested on 82 other cancer centers across Ontario, Canada. Violin plots show AUROCs for predicting symptom deterioration, with each dot representing performance at a single center. AUROC, area under the receiver operating characteristic curve.

The external validation cohort included 12,079 patients who received 77,003 treatments across 82 cancer centers. Compared with the development cohort at PM, a larger proportion of the external validation cohort consisted of long-term residents (78.4% *v* 92.1%) and people from rural areas (2.6% *v* 15.6%; Data Supplement, Table S22). Among the 82 cancer centers, four exhibited larger volumes of treatment sessions than PM. In the external validation cohort, the system achieved an AUROC meta-analyzed across centers ranging from 0.67 (95% CI, 0.66 to 0.68) for anxiety to 0.73 (95% CI, 0.72 to 0.74) for drowsiness. Heterogeneity in AUROC was observed across centers, with I^2^ ranging from 46.4% to 66.9% for six of the nine symptoms (*P* = .004 for dyspnea, *P* < .001 for the rest; Data Supplement, Table S23). AUROC performance varied across cancer centers, with the largest range being 0.32-0.77 for appetite and the smallest being 0.64-0.80 for drowsiness (Data Supplement, Table S24, Fig 4). Center-level attributes were not associated with performance at validation (Data Supplement, Figs S8-S14).

## DISCUSSION

In this study, ML systems predicted future symptom deterioration among people receiving treatment for cancer using routinely collected EHR data. Systems were well calibrated and provided net benefit in decision curve analyses. Alarms from the systems identified patients at increased risk of future worsening symptoms and needing care in the ED. These results suggest that ML systems could identify high-risk patients who could benefit from interventions to prevent future symptoms. During external validation, the performance of the ML systems was heterogeneous across 82 other centers throughout the health care system, which suggests that ML systems may not generalize if deployed across a health care system without monitoring and fine-tuning at individual centers.

These results demonstrate an opportunity to prevent cancer-related symptoms based on personalized risk assessments using ML. Several strategies to prevent cancer-related symptoms exist, but are associated with resource use or costs. For example, the managing cancer and living meaningfully psychological intervention can prevent anxiety and prevention,^34^ and guidelines recommend various strategies to prevent nausea associated with systemic therapy.^11^ Furthermore, the predictable nature of the broad range of cancer-related symptoms evaluated in this study suggests an opportunity to use ML to select patients for studies of novel symptom prevention strategies.

The strengths of this study include the use of routinely collected EHR data, ML modeling, the prediction of various self-reported symptoms, a large training cohort, and an external validation across a health care system. Consequently, this study deepens the understanding of how ML can predict future symptoms beyond previous studies with smaller sample sizes, fewer symptoms, and lack of validation.^12-14,35^ Taken together, these studies support that future symptoms are predictable using ML.

Simulated deployment showed how the frequency of alarms would increase when simultaneously predicting multiple symptoms. More alarms can cause alarm fatigue, missed alarms, burnout on the clinical team, and, ultimately, unsuccessful implementation.^36^ We explored two pragmatic strategies to restrict the overall alarm rate when predicting multiple targets: (1) alarm-based allocation, where each target receives the same number of alarms, and (2) risk-based allocation, where a single risk threshold is used across all targets. These approaches could be extended by upweighting specific targets based on importance. Principled strategies to control alarm fatigue will become increasingly important as more systems are deployed in clinical practice.

Ontario evaluates cancer centers on completion rates of the ESAS,^21,37^ which provided a unique opportunity to study generalization across a health care system.^38^ Despite developing the ML systems at a single large academic cancer center, the overall performance was maintained in an external validation across 82 diverse cancer centers. However, performance varied by cancer center, demonstrating significant heterogeneity, which was unexplained by center-level features. These findings strongly support that ML systems should be monitored and fine-tuned as needed at each center before deployment in clinical care. Solutions should scale efficiently to smaller and potentially under-resourced centers where systems from large centers may not generalize.

This study has several limitations. First, this analysis was retrospective among patients with aerodigestive cancer. Prospective silent deployment in broader patient populations would be required before implementation.^39^ Second, predictive features came from routinely collected tabular EHR data. Additional data modalities, such as remote patient monitoring^40^ or text from clinical reports,^41^ may further improve performance. Third, given the real-world nature of our data, a subset of patients did not complete ESAS questionnaires. We did not observe major differences across patient characteristics based on missingness, and missingness was not associated with system performance at the center level. Nonetheless, how these results would generalize to patients who did not report symptoms cannot be directly answered using available data. Finally, future research should integrate these systems into the clinical workflow along with personalized interventions to prevent future symptoms. Decision curve analysis showed these systems should provide benefit when guiding interventions across a range of cost-benefit trade-offs.

In conclusion, ML systems can predict future symptoms among people with cancer from EHR data, although systems trained at one cancer center may not generalize consistently to others. These results uncover an opportunity to prevent future symptoms using ML-guided personalized preventive care. Scalable solutions to evaluating and fine-tuning ML systems across a health care system will be required for successful implementation.

## Data Availability

A data sharing statement provided by the authors is available with this article at DOI https://doi.org/10.1200/CCI-25-00073. This document used data adapted from the Statistics Canada Postal Code^OM^ Conversion File, which is based on data licensed from Canada Post Corporation, and/or data adapted from the MOH Postal Code Conversion File, which contains data copied under license from Canada Post Corporation and Statistics Canada. Parts of this material are based on data and information compiled and provided by: MOH, CIHI, Ontario Health (OH), and Immigration, Refugees and Citizenship Canada (IRCC). The code is publicly available at https://github.com/ml4oncology/PredUCE.
